# Aurora-A recruitment and centrosomal maturation are regulated by a Golgi-activated pool of Src during G2

**DOI:** 10.1038/ncomms11727

**Published:** 2016-05-31

**Authors:** Maria Luisa Barretta, Daniela Spano, Chiara D'Ambrosio, Romina Ines Cervigni, Andrea Scaloni, Daniela Corda, Antonino Colanzi

**Affiliations:** 1 Institute of Protein Biochemistry (IBP), National Research Council (CNR), Via P. Castellino 111, 80131 Naples, Italy; 2 Proteomics and Mass Spectrometry Laboratory, Institute for the Animal Production System in the Mediterranean Environment, ISPAAM, National Research Council (CNR), Via Argine 1085, 80147 Naples, Italy

## Abstract

The Golgi apparatus is composed of stacks of cisternae laterally connected by tubules to form a ribbon-like structure. At the onset of mitosis, the Golgi ribbon is broken down into discrete stacks, which then undergo further fragmentation. This ribbon cleavage is required for G2/M transition, which thus indicates that a ‘Golgi mitotic checkpoint' couples Golgi inheritance with cell cycle transition. We previously showed that the Golgi-checkpoint regulates the centrosomal recruitment of the mitotic kinase Aurora-A; however, how the Golgi unlinking regulates this recruitment was unknown. Here we show that, in G2, Aurora-A recruitment is promoted by activated Src at the Golgi. Our data provide evidence that Src and Aurora-A interact upon Golgi ribbon fragmentation; Src phosphorylates Aurora-A at tyrosine 148 and this specific phosphorylation is required for Aurora-A localization at the centrosomes. This process, pivotal for centrosome maturation, is a fundamental prerequisite for proper spindle formation and chromosome segregation.

## Abstract (web summary)

The Golgi mitotic checkpoint couples Golgi inheritance with cell cycle transition, and regulates centrosomal recruitment of the mitotic kinase Aurora-A. Here the authors show that upon Golgi ribbon fragmentation in G2, Src phosphorylates Aurora-A at the Golgi, driving its localization to the centrosomes.

## Graphical abstract

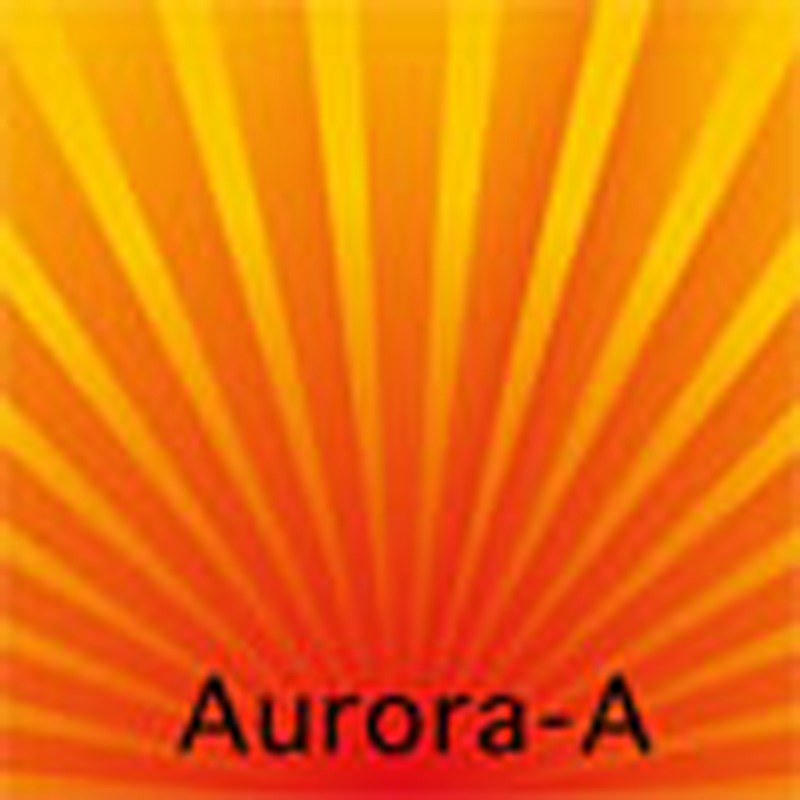

The Golgi apparatus is a major station of the secretory pathway that processes and sorts newly synthesised proteins and lipids during the transport from the endoplasmic reticulum (ER) to the plasma membrane or to the endo-lysosomal system. The Golgi apparatus has been also defined as a ‘carbohydrate factory', for the glycosylation and other post-translational modifications of proteins and lipids, and, finally, it acts as a signalling platform onto which diverse signalling, sorting and cytoskeleton proteins can localize and function^1^. Structurally, the Golgi apparatus is composed of stacks of flat cisternae that are laterally connected by membranous tubular ‘bridges' to form a continuous system, which is referred to as the ‘Golgi ribbon'^2^.

In mammals, the mitotic inheritance of the Golgi apparatus involves its progressive fragmentation, which is a process that can be schematically divided into three main steps: (i) the breakup of the Golgi ribbon into stacks (referred to as unlinking), which occurs in G2-phase and is detectable only using high-resolution imaging techniques^3^; (ii) the fragmentation of these stacks into cisternae (referred to as unstacking), which occurs in prophase; and (iii) finally, the further fragmentation into dispersed fragments and vesicles. Once the dispersed Golgi membranes have been divided between the daughter cells during telophase, they can then reassemble and fuse into a new fully functional Golgi ribbon^2^.

Importantly, inhibition of Golgi ribbon unlinking results in G2-arrest of the cell cycle^4^,^5^,^6^, or in delay of G2/M transition^7^, depending on the experimental approach^8^. This indicates that a Golgi mitotic checkpoint controls the correct inheritance of this organelle^5^,^7^,^9^. We have previously addressed the functional connection between Golgi fragmentation and cell cycle regulation and we have shown that a block in this Golgi unlinking results in a reduced recruitment of Aurora-A kinase to the centrosomes and its impaired activation at the centrosomes during the G2-phase of the cell cycle^10^. Aurora-A has a pivotal role in many centrosome-based processes. Indeed, a complex network of regulatory systems ensures an ordered and spatially restricted activation of Aurora A to control centrosome maturation, entry into mitosis, formation of the bipolar spindle and cytokinesis^11^. Moreover, a Golgi-localized mitotic kinase Myt1 promotes Golgi fragmentation and early entry into mitosis^12^.

Thus, these findings indicate that a sensor-based signalling circuit can monitor the state of (dis)assembly of the Golgi apparatus and convey this information to Aurora-A. However, the composition and the organization of this signalling pathway are not known.

Data from the literature suggest that this sensor-based circuit can be mediated by one or more of the proteins belonging to the non-receptor Src family of tyrosine kinases (SFK_s_) This family comprises nine members (Src, Yes, Fyn, Fgr, Lck, Hck, Blk, Lyn and Frk, divided into three main subfamilies), many of which are co-expressed in cells and can exert redundant functions and/or compensate each other^13^. For instance, ablation of SFK_s_ activity by microinjection of a Src-blocking antibody results in potent G2-arrest^14^; moreover, several roles for SFK_s_ have been described for mitosis-associated events, such as segregation of duplicated centrosomes and spindle formation^15^,^16^. In addition, although the SFK_s_ are mainly known for their transduction of signalling events from the plasma membrane, they also have key roles in signalling events confined to endomembranes. SFK_s_ also regulates signalling events that originate from the Golgi apparatus and that regulate also non-Golgi-based functions, such as extracellular matrix degradation^17^,^18^. Thus, in this study we tested the hypothesis that SFK_s_ mediate the signalling events that operate downstream of the unlinking of the Golgi apparatus, to promote the G2/M transition; due to the functional redundancy between members of the Src family kinases, and the lack of antibodies to identify the specific activated members, we will not differentiate among the single members and henceforth we will refer to the SFK_s_ as Src.

Here we provide evidence that Src is a key element of a signalling circuit that coordinates Golgi unlinking with Aurora-A recruitment to the centrosome. We show that Src is activated at the *trans*-Golgi network (TGN) in coincidence with the G2-specific Golgi unlinking. Moreover, we show that Golgi unlinking controls the activation of Src and the interaction of Src with Aurora-A, thus resulting in the Src-mediated phosphorylation of Aurora-A at Tyr148. This modification in turn promotes Aurora-A recruitment to the centrosome and kinase activity, which are key events in the centrosomal maturation. In conclusion, our findings support the key role of the Golgi apparatus in the regulation of the mitotic entry.

## Results

### Src controls Aurora-A centrosomal recruitment

**Figure 1:**
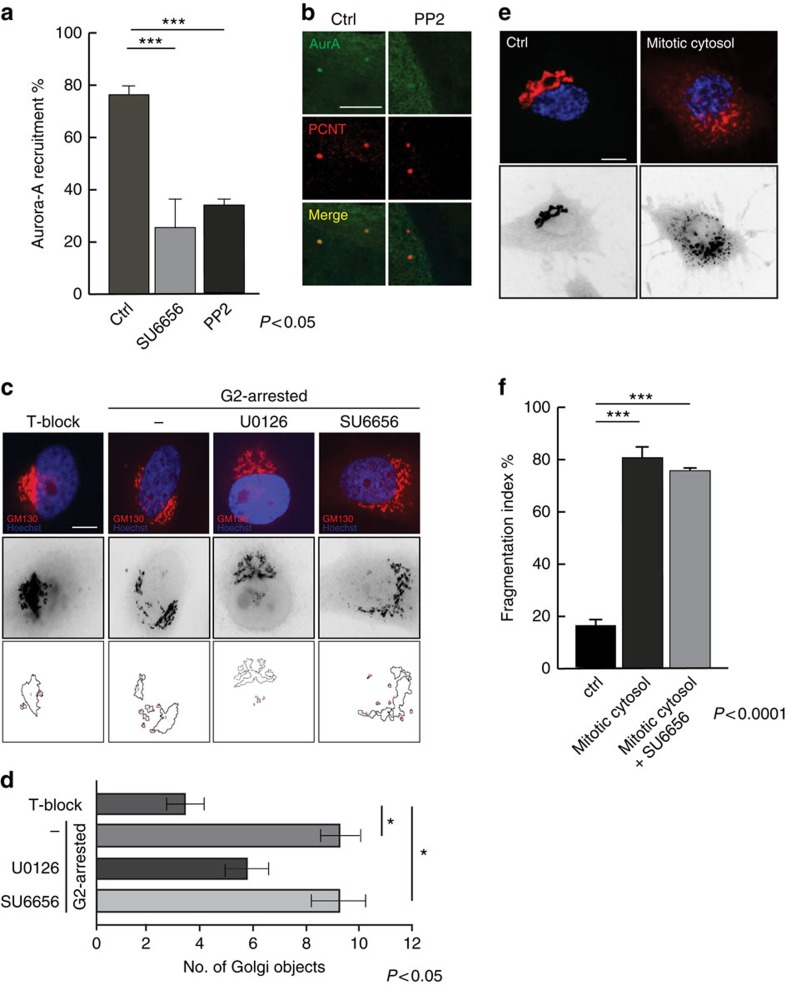
Src-dependent Aurora-A recruitment is a process downstream the Golgi ribbon fragmentation. (**a**) HeLa cells grown on coverslips and arrested in S-phase using double-thymidine block. Two hours before fixing, cells were incubated with 10 μM SU6656 or PP2 or DMSO. After thymidine washout, cells were processed for immunofluorescence analysis with anti-Aurora-A and anti-γ-tubulin antibodies. Graph is the quantification of the percentage of cells positive for Aurora-A at the centrosome as means (±s.d.) from three independent experiments (*n*=200). Two-tailed Student *t*-test was applied to the data (*P* value <0.05) (**b**) Representative images of quantified cells as in **a**. Scale bar, 3 μm. (**c**,**d**) HeLa cells arrested in S-phase with double-thymidine block (T-block) or accumulated in G2 with bis-benzimide for 18 h (G2-arrested), respectively. Where indicated, cells were treated with 80 μM MEK inhibitor (U0126) or DMSO (6 h) or 10 μM SU6656 (2 h). (**c**) Representative images show Hoechst 33342-labelled DNA (blue) and the Golgi complex (red) stained with anti-GM130 antibody. For qualitative analysis of the Golgi, the GM130 staining was inverted using ‘Invert' function of the ImageJ software package. Scale bar, 5 μm. (**d**) Quantification of Golgi linking in the populations in **c** with ImageJ software to apply a threshold and count the above-threshold fluorescent ‘Golgi objects', using the ‘Analyse Particle' function. The analysed cells (*n*=50) were chosen randomly. Data are mean values (±s.e.m.) from three independent experiments. Two-tailed Student's *t-*tests were applied to the data (*P* value<0.05). (**e**,**f**) NRK cells grown on coverslips and arrested in S-phase with 2.5 μg ml^−1^ aphidicolin. After aphidicolin washout, cells were washed for cell cycle progression. Src inhibitor SU6656 was added to the mitotic cytosol before the permeabilization procedure and cells were incubated with mitotic cytosol with SU6656 or DMSO. The Golgi apparatus was labelled with anti-giantin antibody (red). (**e**) Representative images of the cells labelled with anti-giantin (red) for the Golgi and with Hoechst 33342 (blue) for the cell cycle phase. Scale bar, 5 μm. (**f**) Quantification of the extent of fragmentation of the Golgi measured by counting the number of cells (*n*=20) in which the Golgi apparatus was fragmented/dispersed. Data are mean values (±s.d.) from three independent experiments, each carried out in duplicate. Two-tailed Student's *t*-tests were applied to the data (*P* value<0.0001). DMSO, dimethylsulphoxide.

Data from literature provide a strong evidence for a role for Src in the regulation of G2/M transition^14^, but the mechanism of such a regulation is still not completely understood. We asked whether the inhibition of Src could phenocopy the block of Golgi ribbon unlinking in G2 on the reduction of Aurora-A recruitment to the centrosome^10^ as it has been observed upon microinjection of blockers of Golgi fragmentation^10^ or inhibition of ribbon unlinking by chemical inhibitors of MEK (ref. 7) and Jnk2 (ref. 19) (Supplementary Fig. 1a–b). In HeLa cells synchronized for the G2/M enrichment by the double-thymidine S-phase block (Supplementary Fig. 2a), the incubation with the Src inhibitors SU6656 or PP2 caused a strong reduction in the fraction of cells with detectable levels of Aurora-A at the centrosome (Fig. 1a,b). This inhibition was not the consequence of the delayed G2/M transition caused by Src inhibition, as we fixed the samples at a time when most of the cells were in G2 and were positive for Aurora-A at the centrosome^10^.

Src overactivation is known to induce Golgi fragmentation by stimulating dynamin-dependent post-Golgi traffic^18^,^20^. Thus, we asked whether Src inhibition could also prevent the G2-specific Golgi unlinking, as a block of this process can indirectly result in a reduction of Aurora-A recruitment^10^. HeLa cells were either accumulated in S-phase (T-block) or in G2-phase (G2-arrested). These G2-arrested HeLa cells were also treated with U0126, to inhibit MEK activity that is required for the severing of the Golgi ribbon^7^,^21^, and with SU6656, to test the requirement for Src in fragmentation of the Golgi ribbon. The cells were then fixed and processed for confocal microscopy (Fig. 1c, upper panel) to analyse the extent of the ribbon unlinking.

In S-phase-enriched cells, the Golgi membranes were composed of an average of four Golgi ‘objects' (Fig. 1c,d, T-block, lane 1), as previously described^7^,^10^; instead, in G2-arrested cells, the Golgi complex was composed of about 9 ‘objects' (Fig. 1c,d, G2-arrested, lane 2), as expected for G2-arrested cells^7^,^10^. Inhibition of MEK in G2-blocked cells resulted in a decrease in the extent of fragmentation, in line with previous results (Fig. 1c,d, G2-arrested+U0126, lane 3)^7^,^21^. Conversely, inhibition of Src did not reduce Golgi fragmentation (Fig. 1c,d, G2-arrested+SU6656, lane 4).

Thus, we further tested the role of Src in mitotic Golgi fragmentation using an *in vitro* assay^21^. In permeabilized NRK cells incubated with interphase cytosol, the Golgi remained compact and perinuclear (Fig. 1e,f, ctrl). Conversely, in cells incubated with the mitotic cytosol, the Golgi apparatus was fragmented and dispersed^21^ (Fig. 1e,f, mitotic cytosol). Src inhibition in mitotic extracts using SU6656 did not cause any reduction in the fraction of cells with fragmented Golgi (fragmentation index; Fig. 1f, mitotic cytosol+SU6656), thus confirming that Src is not involved in G2-ribbon unlinking and mitotic fragmentation.

### Golgi fragmentation in G2 induces activation of Src

As Src inhibition induces a delay in the G2/M progression^14^ (Supplementary Fig. 2b,c) and an impairment of the recruitment of Aurora-A to the centrosome (Fig. 1a), without affecting the structure of the Golgi apparatus (Fig. 1c,d), one possibility is that a Src-based signalling pathway acts downstream of the Golgi ribbon breakup, which then leads to the recruitment of Aurora-A.

**Figure 2:**
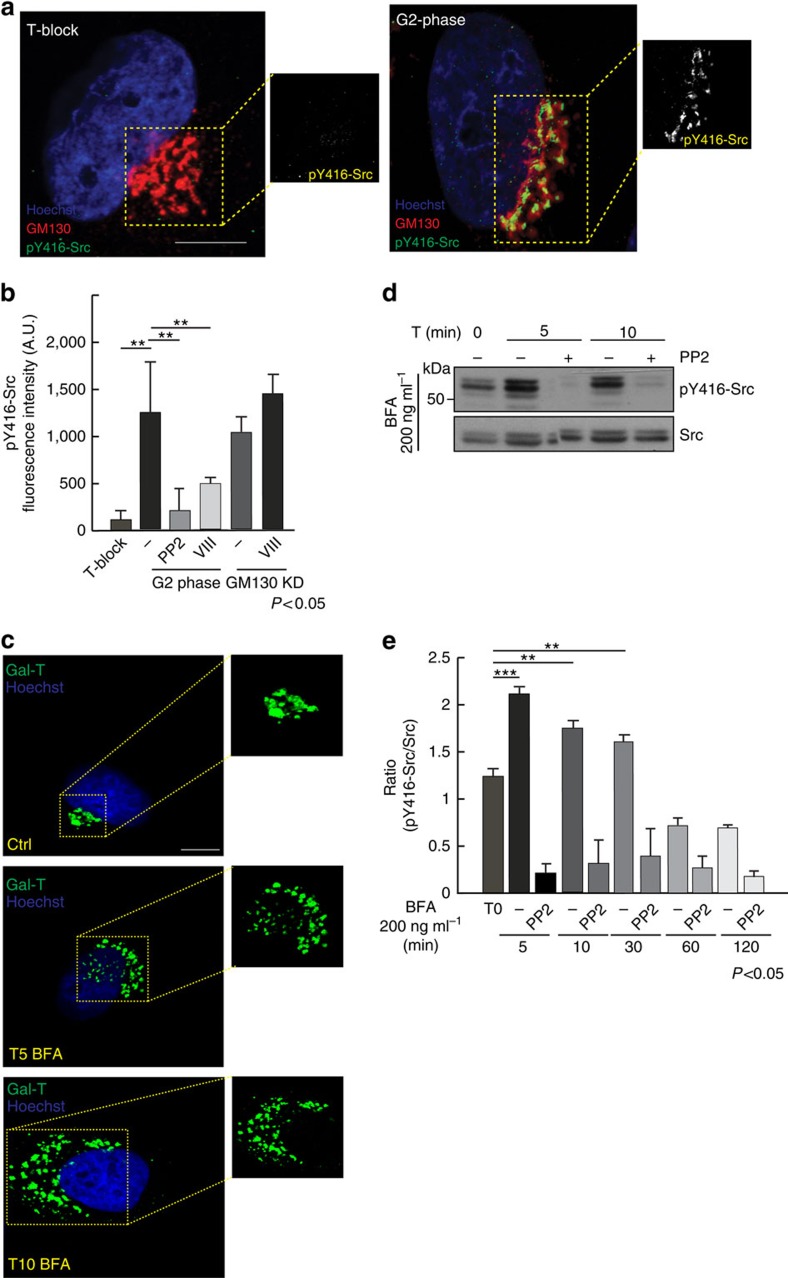
The Golgi ribbon unlinking in G2 induces local activation of Src in HeLa cells. Cells grown on coverslips and processed for immunofluorescence confocal microscopy after S-phase block release. (**a**) Representative images of cells at the indicated cell cycle phase, labelled with anti-pY416-Src (green; insets), GM130 (red) antibodies and Hoechst 33342 (blue) for the nuclei. Scale bar, 4 μm. (**b**) Quantification of cells as in **a** showing fluorescence intensity of pY416-Src (A.U.). For the S-phase block (T-block), cells were treated with DMSO soon after the thymidine-block release; for the other conditions, the cells were incubated with DMSO (−), PP2 (10 μM) or JNKi-VIII (50 μM) for 2 h before fixing (8 h after the S-phase-block release). For GM130 depleted cells (GM130 KD), interference was carried out for 24 h according to the synchronization procedure. GM130 KD cells were treated with vehicle (−) or JNKi-VIII before fixing. All the images analysed were acquired at maximal resolution under fixed-imaging conditions. Equal areas were used to select the Golgi regions and a non-Golgi region with a similar background. Quantification data are mean values (±s.d.) from two independent experiments, each carried out in duplicate. Two-tailed Student's *t*-tests were applied to the data (*n*=50; *P* value <0.05). (**c**) HeLa cells arrested in S-phase using the thymidine block, and, then grown under starving conditions (0.1% foetal calf serum (FCS)) for 1 h to downregulate Src activation because of the growth factors contained in the medium. Cells were then treated with DMSO (−) or with 200ng ml^−1^ BFA for 5, 10, 30, 60 and 120 min in combination with PP2. Representative images of cells grown on coverslips and processed for immunofluorescence to monitor the BFA-induced extent of Golgi fragmentation. The cells were labelled with anti-Gal-T antibody (green; Golgi complex) and Hoechst 33342 for nuclei (blue). Images were acquired at maximal resolution under fixed-imaging conditions. Scale bar, 5 μm. (**d**) After the treatments as in **c**, cells were lysed with a buffer containing protease and phosphatase inhibitors. Equal amounts of clarified cell lysates were loaded onto SDS–PAGE and immune-blotted with anti-pY416-Src and anti-Src antibodies. (**e**) Quantification of **d** expressed as pY416-Src/Src. Two-tailed Student's *t*-tests were applied to the data (*P* value<0.05). A.U. arbitrary unit; DMSO, dimethylsulphoxide.

If this is the case, the fragmentation of the Golgi ribbon during G2 should induce the activation of the kinase Src, which can be monitored by the phosphorylation level of Tyr416 (pY416-Src). Src has been reported to be located not only at the level of the plasma membrane but also in other cellular compartments, including the Golgi apparatus^18^. Synchronized HeLa cells were fixed in coincidence with the thymidine washout (S-phase blocked) or 8 h later (G2-phase). Some of the cells were also treated with the JNK inhibitor VIII (JNKi-VIII), to prevent Golgi ribbon unlinking^19^ or with the Src inhibitor PP2. To reveal active Src at the Golgi apparatus, the cells were washed with a digitonin-containing buffer before fixing and immunolabelling. This permeabilization was necessary for the extraction of the cytoplasmic pool of Src. Representative images of cells stained for the active form of Src are shown in Fig. 2a.

The cells were examined by confocal analysis for a quantitative assessment of the fluorescence intensity associated with active Src, using the staining pattern of GM130 as the reference for the Golgi area. As shown in Fig. 2a,b in S-phase cells (T-block), a faint but detectable level of active Src was associated with the Golgi apparatus. Importantly, the intensity of the Golgi-associated active Src increased during G2-phase. This staining was specific for active Src because it was reduced by the Src inhibitor PP2 (Fig. 2b). Importantly, a block of G2-specific ribbon unlinking by treatment with the JNKi-VIII (ref. 19) strongly reduced the activation of Src at the Golgi (Fig. 2b). To determine whether the activation of Src is directly regulated by Golgi unliking, the Golgi apparatus was forced to be in the form of isolated stacks through siRNA-mediated GM130 depletion^22^ (Fig. 2b), as this condition induces bypass of the Golgi checkpoint^10^. When the Golgi complex was in the form of isolated stacks, the JNKi-VIII did not reduce the levels of active Src (Fig. 2b), thus suggesting that Src is specifically activated by the unlinking process. Interestingly, active Src co-localized with TGN46, a marker of the TGN/late-endosomes, but not with GM130, a marker of the *cis*-Golgi (Supplementary Fig. 3a–b), which suggests that the signalling originates from the TGN. Moreover, we have employed the proximity ligation assay (PLA) to test the association of Src with Golgi markers. Using this approach, interactions between two proteins result in the formation of fluorescent dots in fixed cells^23^. As shown in Supplementary Fig. 3c–f, Src appears to be located at the Golgi complex in association with α-mannosidase-II (ref. 24), independently of the cell cycle phase, thus suggesting that its activation, and not its recruitment, is the step regulated by Golgi fragmentation.

These results indicated that the Golgi fragmentation correlates with the activation of Src at the Golgi apparatus. To address this aspect by an independent approach, we monitored the activation of Src by western blotting of total cell lysates using the anti-pY416-Src antibody. To induce Golgi fragmentation, HeLa cells were synchronized in S-phase and treated for a time from 5 to 120 min with brefeldin-A (BFA), a fungal toxin that causes disassembly of the Golgi apparatus^25^. The cells were then processed for both confocal analysis, to monitor the Golgi structure and western blotting, to verify Src activation. Under these conditions, a short treatment with BFA resulted in the loss of the ribbon-like organization of the Golgi (Fig. 2c), and this correlated with Src activation, as revealed by western blotting (Fig. 2d,e). Activation of Src peaked after 5 min incubation with BFA, and was specifically inhibited by the Src inhibitor PP2 (Fig. 2d,e). After 30 min of incubation with BFA, the Golgi apparatus is completely dissolved and redistributed into the ER^26^. Thus, it is plausible that the machinery responsible for Src activation has been disrupted after this time point, resulting in basal levels of active Src (Fig. 2e).

Altogether, these data suggest that the Golgi apparatus acts as a signalling hub to monitor Golgi unlinking and to induce compartmentalized Src activation^27^, which could be an important determinant in functionally linking the Golgi breakdown to Aurora-A.

### Golgi unlinking induces Src-Aurora-A interaction

**Figure 3:**
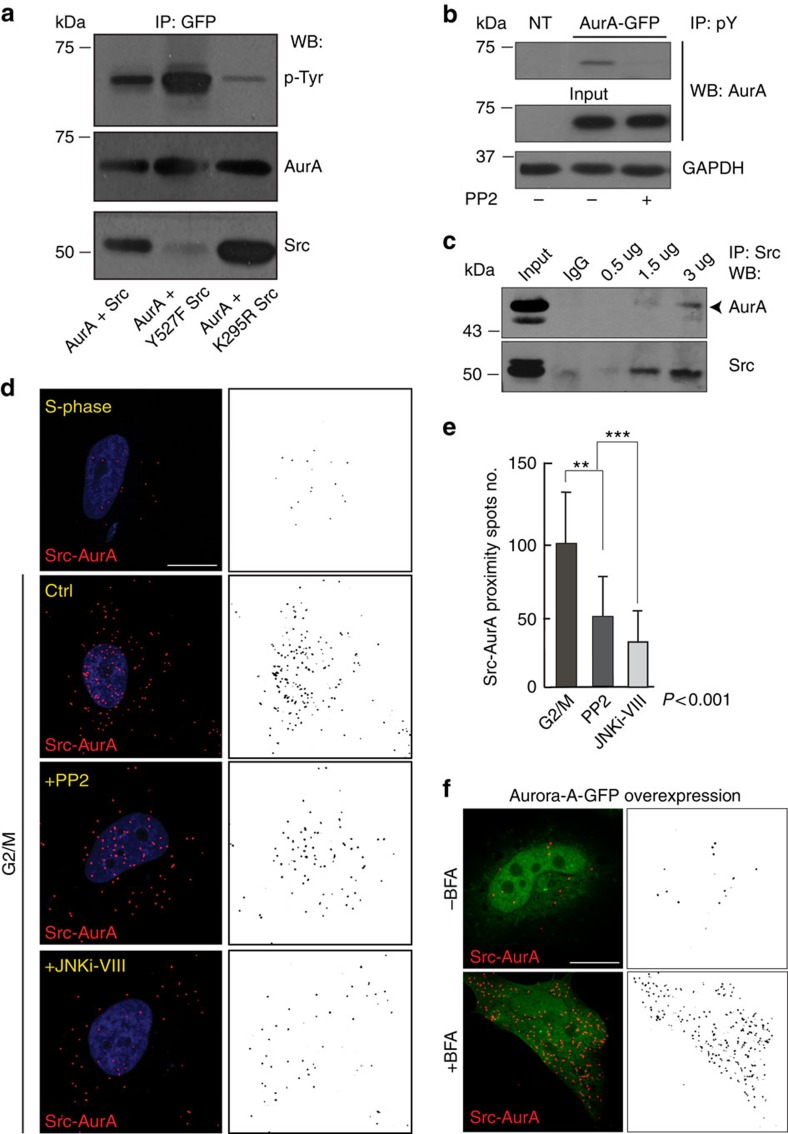
Src physically associates to Aurora-A and this interaction is modulated by inhibition of Golgi fragmentation. (**a**) HeLa cells arrested in S-phase with double-thymidine block and transfected for 24 h with Aurora-A-GFP together with wt, constitutively active (Y527F) or kinase dead (K295R) Src mutants after the first release from thymidine. Immunoprecipitation was performed from G2-enriched population by incubating 1 mg of clarified cell lysates with anti-GFP antibody, O/N at 4 °C. Co-precipitated proteins were separated onto 8% SDS–PAGE gel and the extent of tyrosine phosphorylation was analysed by western blotting. (**b**) The phosphorylation on Aurora-A was examined also by immunoprecipitation with anti-phospho-tyrosine agarose-conjugate antibody after 24 h of overexpression of Aurora-A-GFP. Where indicated, 10 μM PP2 was used for 2 h before protein extraction to assess the specificity of the Src-mediated phosphorylation. (**c**) HeLa cells synchronized with double-thymidine block, and lysed 10 h after the second thymidine washout. Five hundred microgram of total cells lysates were incubated with different amounts of anti-Src antibody (0.5, 1.5 or 3 μg mg^−1^ of total proteins) or with anti-rabbit pre-immune IgG_s_ (3 μg mg^−1^ of total proteins), O/N at 4 °C. After the incubation, the immune complexes were precipitated using protein A-Sepharose and SDS–PAGE was performed. The co-precipitated proteins were revealed with anti-Src and anti-Aurora-A antibodies. (**d**,**e**) HeLa cells grown on coverslips and arrested in S-phase with double-thymidine block. Two hours before fixing, cells were incubated with 10 μM PP2 or 50 μM JNKi-VIII. After 8 h without thymidine, the cells were processed under confocal microscopy with 2 mg ml^−1^ Hoechst 33342 for visualization of nuclei (blue), and subjected to PLA with anti-Aurora-A and anti-Src antibodies. The interactions were expressed as the numbers of red dots each of which indicates the proximity of Src and Aurora-A. All images analysed were acquired at maximal resolution under fixed-imaging conditions. (**d**) Representative images of PLA as above described. Scale bar, 5 μm. (**e**) The quantification of cells treated as in **d**. Data are means (±s.d.) from three different experiments. Two-tailed Student's *t*-tests were applied to the data (*n*=50; *P*<0.001). (**f**) Representative images of PLA between endogenous Src and overexpressed Aurora-A-GFP was performed on synchronized HeLa cells treated with 200 ng ml^−1^ BFA for 2 h (+BFA). Scale bar, 5 μm.

We next examined whether Golgi-dependent Src activation and Src-dependent Aurora-A recruitment are functionally linked. Thus, HeLa cells enriched in the G2-phase were co-transfected with vectors for the expression of Aurora-A-GFP and the untagged versions of the wild-type (wt), constitutively active (Y527F) and kinase-dead (K295R) forms of Src. Immunoprecipitation of Aurora-A-GFP revealed that Aurora-A and Src co-precipitated (Fig. 3a, lower panel). The inactive form of Src (K295R) was bound more efficiently to Aurora-A-GFP with respect to wt-Src or the Y527F-Src mutant (Fig. 3a, lower panel), thus suggesting that the inactive form of Src would be released more slowly from its substrate. Moreover, the possibility that Aurora-A is phosphorylated in living cells by Src was examined. The level of tyrosine phosphorylation of Aurora-A was higher in the lysates that overexpressed wt-Src and constitutively active Y527F-Src when compared with the cells that overexpressed the kinase-dead mutant K295R-Src (Fig. 3a, upper panel).

To further test this finding, HeLa cells were synchronized at the G1/S boundary and lysed 8 h after release from the S-phase (G2-enrichment). Some of the cells were transfected with Aurora-A-GFP and treated with PP2. The proteins phosphorylated at tyrosine were immunoprecipitated using an anti-phospho-tyrosine antibody. As shown in Fig. 3b, Aurora-A was immunoprecipitated under control conditions, but was not immunoprecipitated in the presence of PP2, confirming that Aurora-A is phosphorylated at tyrosine(s) by Src.

In addition, immunoprecipitation of the endogenous proteins in synchronized HeLa cells, showed that endogenous Src and Aurora-A co-precipitated (Fig. 3c). To measure a possible modulation of that interaction quantitatively, we used the PLA as the method of choice to measure variations in the association between endogenous Aurora-A and Src. As confirmation of the cell cycle-dependent interaction between the endogenous proteins, the cells incubated with both anti-Aurora-A and anti-Src antibodies showed a low number of spots in S-phase (Fig. 3d—S-phase) and, as expected, a large number of red dot signals were detected in G2-phase, (Fig. 3d,e—ctrl). The treatment with the Src inhibitor PP2 caused a strong reduction in the number of red dots^28^ (Fig. 3d,e—PP2), probably because PP2 binds to a deep pocket near the ATP-binding cleft of the Src family kinases^29^, thus causing a steric hindrance that can limit the interaction between Src and Aurora-A.

Strikingly, a block of the Golgi-unlinking in G2-phase using JNKi-VIII prevented the association between Aurora-A and Src, in line with our proposal that Src activation signals Golgi unlinking to Aurora-A (Fig. 3d,e—JNKi-VIII). Moreover, in cells blocked in S-phase and transfected with Aurora-A-GFP, as the endogenous Aurora-A protein content is undetectable in this phase, the fragmentation of the Golgi complex with BFA strongly enhanced Src-Aurora-A interaction (Fig. 3f), and thus further confirming that Src-Aurora-A interaction is promoted by Golgi fragmentation.

### Src phosphorylates Aurora-A to induce centrosome maturation

**Figure 4:**
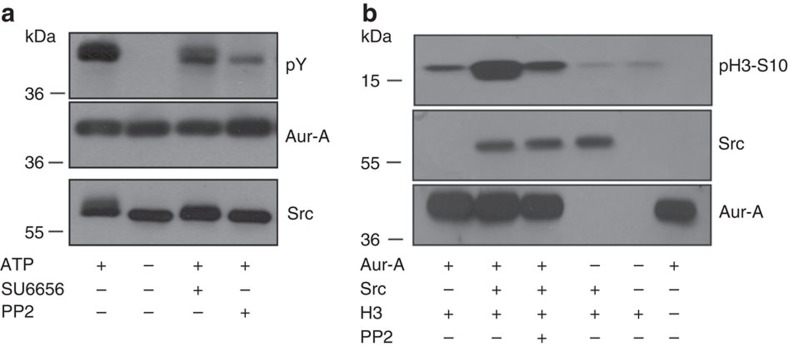
Src phosphorylates Aurora-A, thus increasing its catalytic activity towards an *in vitro* substrate. (**a**) Representative western blotting of an *in vitro* non-radioactive reaction carried out using recombinant purified GST-Src and Aurora-A. When indicated, 50 μM PP2 or 50 μM SU6656 were pre-incubated with GST-Src for 10 min before the addition of Aurora-A. The extent of tyrosine phosphorylation of Aurora-A was analysed using an anti-phospho-tyrosine antibody. (**b**) A double *in vitro* kinase assay was performed using recombinant His-tagged Histone-H3 as a substrate of Aurora-A, which was added to the reaction for 45 min. Where indicated, PP2 was pre-incubated with GST-Src for 10 min before the addition of Aurora-A. The catalytic activity of Aurora-A was measured as extent of pSer10-Histone-H3. Representative western blot images of five different experiments.

To assess the functional relevance of Src-Aurora-A interaction, *in vitro* kinase assays were performed using recombinant GST-Src and untagged Aurora-A, and the extent of Aurora-A phosphorylation was determined using an anti-phospho-tyrosine antibody. Aurora-A was phosphorylated by the kinase Src also *in vitro*, and the modification was almost absent if the Src catalytic activity was impaired by the Src inhibitors SU6656 or PP2 (Fig. 4a).

Next, we analysed Aurora-A activation, using a two-step kinase assay with Src and Aurora-A in the first step, and by subsequent incubation of the recombinant proteins with Histone-H3, a well-validated *in-vitro* Aurora-A substrate that is phosphorylated at Ser10 (ref. 30). Aurora-A-mediated phosphorylation of Histone-H3 on Ser10 was markedly increased when Src was present in the reaction, and was reduced when the catalytic activity of Src was inhibited by PP2 (Fig. 4b). In addition, we did not observe any enhancement of Aurora-A auto-activatory phosphorylation using the antibody that recognizes phosphorylated Thr288, suggesting that the catalytic activity of Aurora-A can be modulated even in the absence of phosphorylation at Thr288 (refs 31, 32).

**Figure 5:**
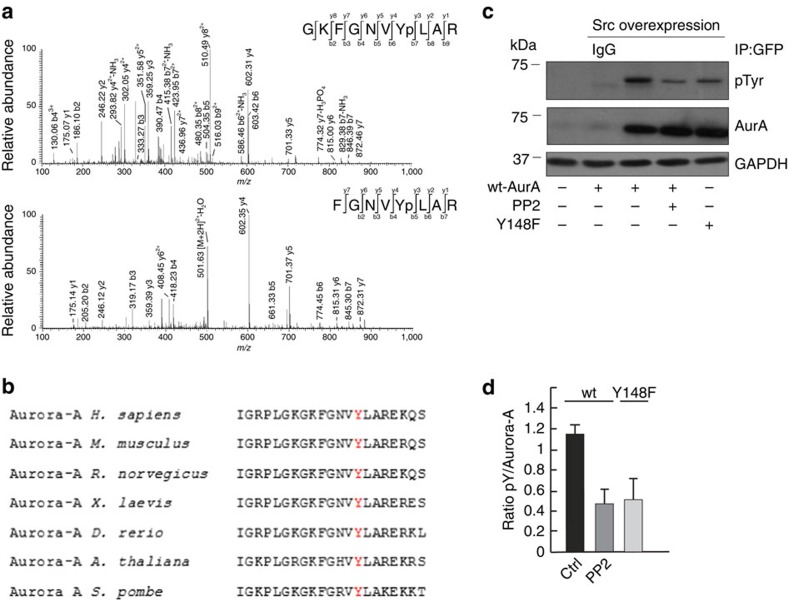
The mutant Aurora-A-Y148F showed an impaired phosphorylation. (**a**) Phosphorylation of Aurora-A at Tyr148 as determined by mass spectrometry analysis. CID fragmentation spectra of two modified peptides bearing the phosphorylation at Tyr148. (upper) MS/MS of the triply charged phosphopeptide (142–151) GKFGNVYpLAR (*m/z* 402.76, MH^+^ equal to 1206.27). (lower) MS/MS of the doubly charged phosphopeptide (144–151) FGNVYLAR (*m/z* 510.74, MH^+^ equal to 1,020.46). (**b**) Alignment of the sequences surrounding human Aurora-A Tyr148 from various species. Y148 site is shown in red. (**c**) HeLa cells were arrested in S-phase using the double-thymidine block. After the first release from thymidine, cells were transfected for 24 h with wt-Aurora-A-GFP or Aurora-A-Y148F-GFP in combination with wt-Src. 2 h before lysis, cells were treated with 10 μM PP2 where indicated. Immunoprecipitation was performed by incubating 1 mg of clarified cell lysates using an anti-GFP antibody, O/N at 4 °C. The precipitated proteins were separated onto 8% SDS–PAGE gels and the extent of tyrosine phosphorylation was analysed by western blotting using an anti-phospho-tyrosine antibody. Representative western blotting of two different experiments. (**d**) Quantification of **c** expressed as pY/Aurora-A. Data are means (±s.e.m.) of two different experiments.

To identify the phosphorylated tyrosine residue(s) on Aurora-A, we performed a phosphoproteomic analysis of the recombinant Aurora-A incubated with Src, which showed that only Tyr148 is phosphorylated under our experimental conditions (Fig. 5a). This residue, which is evolutionary conserved from yeast to mammals (Fig. 5b), has been found to be phosphorylated by a previous phosphoproteomic study^33^, but its physiological role has never been assessed. To determine whether Tyr148 of Aurora-A is modified also *in vivo*, we produced an Aurora-A mutant containing Tyr148 mutated into phenylalanine (Y148F) and performed immunoprecipitation experiments to analyse the extent of phosphorylation. HeLa cells were synchronized by the double-thymidine treatment and co-transfected with vectors for the expression of wt-Aurora-A-GFP or the phospho-depleted mutant (Aurora-A-Y148F-GFP), together with an untagged version of wt-Src. After 24 h of transfection, wt-Aurora-A-GFP and the mutant counterpart Y148F were immunoprecipitated and the degree of phosphorylation was determined by western blotting using an anti-phospho-tyrosine antibody. As shown in Fig. 5c,d, the phosphorylation of wt-Aurora-A was reduced in the presence of the Src inhibitor PP2. An analogous-reduced phosphorylation degree was observed for the Y148F-Aurora-A phospho-depleted mutant, which showed a background level of phosphorylation that was comparable to that of the wt protein where the Src catalytic activity was blocked by PP2 (Fig. 5c,d).

**Figure 6:**
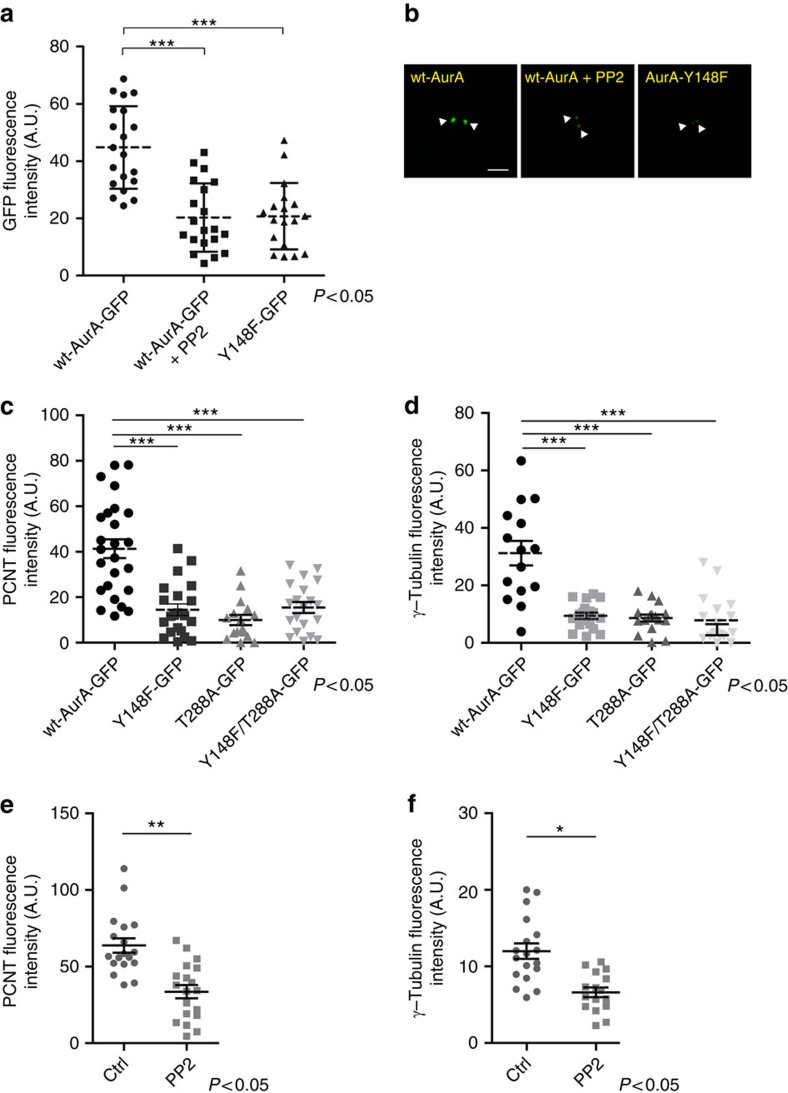
The unphosphorylatable Aurora-A at Y148 fails to recruit to the centrosome and impairs centrosomal maturation. (**a**) Synchronized HeLa cells were transfected for 24 h with wt-Aurora-A or Aurora-A-Y148F tagged with a GFP reporter. Where indicated, cells overexpressing wt-Aurora-A were also treated with 10 μM PP2 for 2 h before fixing. Cells were processed for immunofluorescence to measure the fluorescence intensity of the recruited Aurora-A-GFP, and an anti-γ-tubulin antibody was used as reference for the centrosome (A.U.). All images for the quantification were acquired at maximal resolution, under fixed-imaging conditions. Equal circle areas were used to select the centrosomes regions and a non-centrosome region with a similar background. Quantification data are means (±s.d.) from three independent experiments. Two-tailed Student's *t*-tests were applied to the data (*P*<0.05). (**b**) Representative images of the cells quantified in **a**. Scale bar, 2 μm. (**c**,**d**) Synchronized HeLa cells were co-transfected for a short time period (9 h) with Aurora-A siRNA and siRNA-resistant wt Aurora-A-GFP, Aurora-A-Y148F-GFP, Aurora-A-T288A-GFP or a vector carrying both the mutations (Y148F/T288A). GFP-positive cells were analysed to measure the fluorescence intensity of the recruited PCNT (**c**) or γ-tubulin (**d**) to the centrosome. The images acquisition and the quantifications were performed as described in **a**. Quantification data are the mean values (±s.d.) from three independent experiments. Two-tailed Student's *t*-tests were applied to the data (for PCNT *P*<0.0001; for γ-tubulin *P* value=0.0002). Scale bars, 5 μM. (**e**,**f**) Synchronized HeLa cells were incubated for 2 h with 10 μM of the Src inhibitor PP2 and then processed for immunofluorescence analysis as described in **a**. A.U., arbitrary units.

Next, to examine the functional role of this phosphorylation, G2-enriched HeLa cells were transfected with wt-Aurora-A-GFP,the phospho-depleted (Y148F) and the phospho-mimetic GFP-mutants (Y148D). The GFP-tagged proteins were immunoprecipitated and tested in an *in vitro* kinase assay. As expected, the phospho-depleted mutant protein was less active compared with the wt counterpart (Supplementary Fig. 4a). In addition, also the phospho-mimetic mutant was inactive (Supplementary Fig. 4a). This is not surprising because while phenylalanine can well mimic the structure of a non-phosphorylatable tyrosine, aspartate, being much shorter than phosphotyrosine, and lacking the aromatic ring, should not properly mimic tyrosine replacement, thus not behaving as a phosphorylated residue^34^. Importantly, as a further evidence of the functional role of this phosphorylation, the phospho-depleted mutant of Aurora-A showed a reduced association with the centrosome, which was comparable with the reduced centrosomal recruitment caused by Src inhibition with PP2 (Fig. 6a,b), and in line with the results showed in Fig. 1a. The residual centrosomal recruitment of Aurora-A-Y148F-GFP mutant is likely related to a Src-independent event. In line with the lack of catalytic activity, also Aurora-A-Y148D-GFP fails to be recruited to the centrosome (Supplementary Fig. 4b).

Next, we focused on centrosome maturation, which is one of the key Aurora-A-dependent preparatory events for mitotic ingression and proper chromosomal segregation. Although the centrosomal maturation occurs via a still debated mechanism, it is the result of the recruitment of long coiled-coiled centrosomal proteins, such as Cep192, pericentrin (PCNT), and Cep215 (also called Cdk5-Rap2)^35^, which form a centrosomal matrix that acts as a scaffold for the recruitment and activation of the mitotic kinases and for the recruitment of microtubules (MT) -nucleating and organizing factors^36^. Among them, the most critical is the γ-tubulin ring complex (γ-TuRC), which serves as a MT-nucleating pole. A timely and ordered centrosomal maturation is pivotal for the formation of the spindle and for chromosome segregation. Any imbalance of this process is at the basis of the chromosomal instability.

Given that Aurora-A phospho-depleted mutant (Y148F) showed a reduced centrosomal localization (Fig. 6a,b), we tested whether the overall process of centrosomal maturation, of which Aurora-A is part of 37, was compromised, which could then affect the entry into prophase. Synchronized HeLa cells were depleted of endogenous Aurora-A and transfected for 9 h with siRNA-resistant constructs (Supplementary Fig. 5a) for the expression of wt-Aurora-A-GFP and the phospho-depleted GFP-mutant (Y148F). In addition, to compare the relevance of the newly identified phosphorylated site (Tyr148) with the well-described Thr288, we generated and tested a siRNA-resistant phospho-depleted T288A-GFP and a double siRNA-resistant Aurora-A-Y148F/T288A-GFP mutant. The cells were fixed when they were mostly in G2 and examined by confocal microscopy for quantitative measure of the levels of centrosome-associated PCNT and γ-tubulin. As shown in Fig. 6c,d and Supplementary Fig. 5b,c, cells overexpressing the mutant Aurora-A-Y148F-GFP, showed a strongly reduced centrosomal staining of both PCNT and γ-tubulin, thus indicating a strong impairment of the centrosomal maturation process. This maturation event was also analysed in G2-enriched cells silenced for endogenous Aurora-A and overexpressing the siRNA-resistant phospho-depleted (T288A) and double (Y148F/T288A) mutants. As shown in Fig. 6c,d, both the mutants affected the recruitment of PCNT and γ-tubulin. Thus, these data indicate that the phosphorylation of both Y148 and T288 are necessary for proper centrosome maturation. Moreover, and even in the presence of the endogenous protein, Aurora-A-T288A-GFP and Aurora-A-Y148F/T288A-GFP mutants showed a reduced centrosomal recruitment compared with the wt and similarly to the Aurora-A-Y148F protein (Fig. 6a and Supplementary Fig. 5d), with any additive effect observed for the double point mutant. As expected, the defective centrosomal maturation caused a strong impairment of cell entry into M-phase (Supplementary Fig. 5e). Finally, and in line with an important role of Src, its inhibition with PP2 impaired the recruitment of both PCNT and γ-tubulin, as revealed by the quantitative fluorescence measurement of the two proteins (Fig. 6e,f).

Collectively, our data show that Tyr148, phosphorylated by Src, is crucial for the timely targeting of Aurora-A to the centrosome and to regulate its maturation, which in turn, is a prerequisite for orderly spindle formation and for chromosome segregation.

## Discussion

Precise spatio-temporal localization and activation of mitotic kinases is crucial for the accurate sequential organization of mitotic events. In this study, we have built upon our previous findings that showed that Golgi ribbon unlinking is required for the centrosomal recruitment of the mitotic kinase Aurora-A, which is a key regulator of G2/M transition, centrosome maturation and spindle formation^10^,^38^.

**Figure 7:**
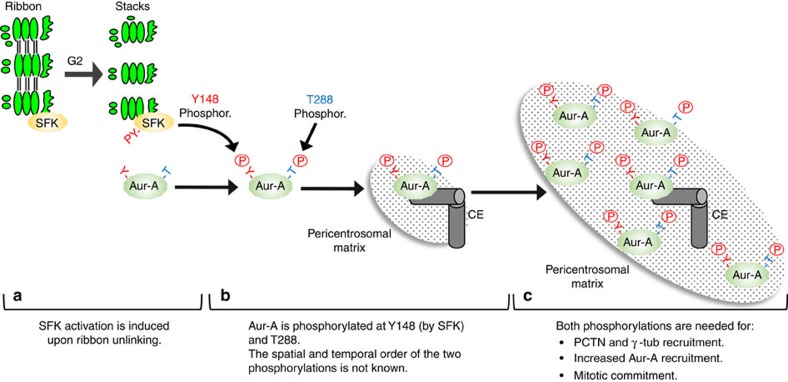
Aurora-A is an integrator of p-Tyr and p-Thr/Ser-based signalling to regulate centrosome maturation and mitotic entry. (**a**) Cleavage of the Golgi ribbon into stacks during the G2-phase of cell cycle induces the activation of a Golgi-based pool of SFK_s_ and, subsequently, the interaction of SFK_s_ with Aurora-A. (**b**) Golgi-activated SFK_s_ then phosphorylates Aurora A in Y148 (Y−); this phosphorylation results in increased Aurora-A kinase activity and centrosomal recruitment. Whether Aurora is phosphorylated at the centrosome or in the cytoplasm by SFK_s_ is not known. The additional phosphorylation of T288 in the activation loop (−T) by either *trans*- or auto-phosphorylations triggers a complex signalling cascade (**c**) that allows the recruitment of additional Aurora-A, PCTN and γ-tubulin for full centrosome maturation and G2/M transition. Aur-A, Aurora-A; CE, centrosome.

Here we have revealed a key element of the signalling pathway that connects Golgi fragmentation to Aurora-A recruitment. Co-immunoprecipitation experiments and PLA showed that a member of the Src family kinases interacts with Aurora-A during G2/M, and that this interaction depends of the state of dis(assembly) of the Golgi ribbon. We show that Src phosphorylates Aurora-A at Tyr148, and that this phosphorylation increases the kinase activity of Aurora-A. Finally, we find that this phosphorylation is crucial for centrosomal maturation. Centrosome maturation involves the recruitment of pericentriolar material components (PCM), such as Cep192/SPD-2, PCNT/PLP, and Cep215 (also called Cdk5Rap2)/Cnn and of an increased MT-nucleating activity^35^. The mechanism and regulation of the assembly of the PCM remains enigmatic; nevertheless, our findings suggest that Aurora-A works as an integrator of different stimuli, as those conveyed on Y148 and T288, to trigger the complex series of events that underlay the assembly of the PCM^35^ (Fig. 7). Whether Aurora is phosphorylated at the centrosome or in the cytoplasm is not known. A possible interpretation of our data is that the reduced level of Aurora-A at the centrosome, which is seen after a block of Golgi fragmentation, is not indicative of reduced affinity for a scaffold but is instead the direct consequence of reduced growth of PCM proteins as reported in the model proposed in Fig. 7.

Aurora-A is a proto-oncogene that is overexpressed in several human cancers and is required at several stages as cells progress towards and through mitosis. Aurora-A levels increase in G2-phase, when the protein is targeted to the centrosome through mechanisms that are only partially understood. Most probably, different scaffolds regulate the recruitment to the centrosome for different specific functions where, according to the classical view, Aurora-A is activated upon phosphorylation at Thr288. Aurora-A activity is protected by the action of phosphatases through the binding of Aurora-A itself to scaffold proteins like HEF1 and Ajuba, which relocate from the focal adhesion to the centrosome during G2/M transition, thus revealing a ‘point of dialogue' between focal adhesions and the centrosome^39^. Given the known functional association of the Golgi apparatus with the centrosome^40^, it is reasonable that a similar interplay between the Golgi membranes and the centrosome coordinate the severing of the Golgi ribbon, with the mechanisms underlying G2-specific activation of Aurora-A at the centrosome. Moreover, our identification of this Src-Aurora-A signalling axis supports the current view that the Golgi apparatus is a central hub where cargo sorting/processing, basic metabolism, signalling and cell cycle decisional processes converge^1^. Connected to this hypothesis, upon mitotic entry the Golgi-associated protein p115 is dissociated from the Golgi and this favours the interaction of the golgin GM130 with importin-α^41^. This interaction leads to sequestration of importin-α and liberates the spindle assembly factor TPX2, which can then activate Aurora-A kinase and stimulate MT nucleation at the centrosome. Thus, the Golgi fragmentation can regulate two subsequent key spindle-based events through Aurora-A: the maturation of the centrosome during G2 and spindle formation at the onset of mitosis^41^.

Our findings have also revealed that Src-mediated phosphorylation of Aurora-A at Tyr148 increases its catalytic activity. Related to this, recent findings on the mechanisms of Aurora-A activation have provided evidence that pT288 is not an accurate reporter of Aurora-A activity. Reboutier *et al*.^31^ proposed that phosphorylation at residues other than Thr288 can induce profound changes in the conformation of Aurora-A, which would increase its catalytic activity. Moreover, Dodson and Bayliss^32^ proposed a revised and dynamic model of Aurora-A activation at the spindle, suggesting that Aurora-A activity is increased 15-fold after the binding to the partner protein TPX2. Similarly, and in line with the latter hypothesis, our results indicate that Src can also increase the *in vitro* catalytic activity of Aurora-A, perhaps through an allosteric mechanism.

The reduction of PCNT recruitment upon Aurora-A-Y148F overexpression represents an additional important finding. Through its ability to serve as a scaffold for anchoring several centrosomal proteins, PCNT is involved in key centrosome functions, including G_1_/S and G_2_/M-transitions, centrosomal maturation and mitotic spindle organization/orientation^42^. For example, recent studies demonstrated that the absence of PCNT at the centrosome is associated with microcephalic osteodysplastic primordial dwarfism type-II (MOPD-II), a human autosomal recessive genetic disorder^43^. Thus, our findings open the possibility that the genetic disorder MOPD-II can also rely on Aurora-A mutations. Further *in vitro* functional investigations and genetic studies on patients cohorts are needed to address this question.

The next important questions are to identify the precise member of the Src family kinases involved in Aurora-A phosphorylation and how the cleavage of the Golgi ribbon is sensed and signalled to Src. Key players in keeping the ribbon structure are the Golgi stacking factors GRASP55 and GRASP65. They have a pro-ribbon function that is reverted by specific phosphorylations. GRASP55 is phosphorylated by the RAF/MEK/ERK kinase module^40^; GRASP65 is phosphorylated in Ser277 by Jnk2 (ref. 19) and by Plk1 in Ser189 (ref. 44). Conversely, the fission-inducing protein BARS^45^ is actively involved in the cleavage of the membranous tubules connecting the Golgi stacks^3^. Src is a non-receptor tyrosine kinase with known roles in the transduction of signalling cascades that arise from receptors at the plasma membrane^13^. It has also been shown that Src co-localizes with Golgi markers and is present in Golgi-enriched membrane fractions, which suggested an involvement of Src in the regulation of some of the many Golgi functions^46^. Indeed, Src is activated at the Golgi apparatus by the arrival of cargoes from the ER^18^ in a mechanism that involves G_α_q (ref. 47) and is required for both intra-Golgi and post-Golgi traffic^18^,^47^. This event appears to be not related to Src-activation upon Golgi fragmentation as it is confined to a Golgi compartment different from the TGN. Src can also control post-Golgi traffic through phosphorylation of dynamin2, which is required for Golgi to plasma membrane delivery of cargo^20^. It is unlikely that Src modulates Aurora-A as a consequence of the regulation of post-Golgi trafficking^20^ because we excluded any active involvement of Src in the cleavage of the ribbon and we assessed that Src activation becomes independent of JNK when the Golgi complex is forced to be in the form of mini-stacks, which are traffic competent.

Most relevant to the present study, Src has been shown to be involved in G2/M transition of the cell cycle in fibroblasts^14^, and more generally, there is a plethora of indications that supports a key role for Src in the regulation of the cell cycle^48^. As suggested by the present study, and in line with literature data, the localisation of Src in different subcellular cytoplasmic compartments provides a means of spatially controlling these strong and promiscuous protein kinases. This in turn would guarantee that activation occurs only at the correct location and/or in the correct compartment, thereby conferring functional specificity and ensuring cell viability.

As mentioned for Aurora-A, also Src has a well-established role in tumorigenesis, supporting tumour growth, invasion and metastasis^49^,^50^. It has been reported that, in cancer cell models, the contemporary inhibition of both Src and Aurora-A kinases led to the death of those population of cells that have undergone defective mitoses and failed to re-attach to the substrate^51^. With respect to this, the identification of this new Golgi-derived signalling network based on the Src-Aurora-A axis that controls pre-mitotic (G2/M transition) and post-mitotic^51^ events highlights the importance of new therapeutic strategies aimed at targeting both these proteins in cancer cells. Moreover, many cancer cells lose the compact ribbon-like structure and display a fragmented organization of the Golgi complex^52^. Given the oncogenic potential of Src, it is reasonable to speculate that, as occurs under physiological conditions of Golgi fragmentation in G2, cancer cells with a fragmented Golgi complex continuously turn on the Golgi-dependent signalling pathway required for Src activation thus stimulating uncontrolled cell proliferation and favouring genomic instability.

In conclusion, the data presented herein identify the kinase Src as a downstream component of the molecular machinery that is activated upon Golgi ribbon breakup, at the onset of mitosis. This specific and compartimentalized Src activation regulates the process of centrosome maturation through phosphorylation at Tyr148 and, probably, in coordination with phosphorylation of T288, thus promoting the entry into mitosis and the proper chromosome segregation.

## Methods

### Cell culture and cell cycle synchronization

NRK and HeLa cells were from the American Tissue Type Collection (ATTC, USA) and were grown in DMEM and MEM, respectively (GIBCO), supplemented with 10% fetal calf serum (Biochrom), 1% MEM non-essential amino-acid solution, 1% L-glutamine, 1 U ml^−1^ penicillin and 50 μg ml^−1^ streptomycin (all Invitrogen). Cells were grown under a controlled atmosphere in the presence of 5% CO_2_ at 37 °C. For the cell cycle synchronization of HeLa cells, the population was treated twice O/N with 2 mM thymidine; after each incubation with thymidine, cells were washed and grown in complete medium O/D. For the cell cycle synchronization of NRK cells, the population was incubated with aphidicolin O/N and the day after cells were released from the aphidicolin block. The G2/M phase of NRK and HeLa cells was reached after 8 h from the washout.

### Flow cytometry

Trypsinized HeLa cells were pelleted, washed in cold PBS, and resuspended in ice-cold ethanol while vortexing. The cells were incubated O/N at 4 °C. The next day, the ethanol was removed by centrifugation and the cells were washed in cold PBS and incubated with 50 μg ml^−1^ propidium iodide (Invitrogen) for 30 min in the presence of RNAse (Sigma). The cells were then analysed using Becton Dickinson FACSCantoA instrument (BD). The data were plotted with Diva software, with 20,000 events analysed for each sample.

### Cell transfection and RNA interference

HeLa cells were transfected with the TransIT-LT1 Transfection Reagent (Mirus), according to the manufacturer's instructions. The cDNA of full-length Aurora-A fused to GFP was a kind gift from Dr Stefano Ferrari (Institute of Molecular Cancer Research, University of Zurich, Zurich, Switzerland). The cDNA of wt-Src, Y527F-Src and K295R-Src were a generous gift from Dr Gabriella Castoria (Seconda Università Degli Studi di Napoli—SUN, Italy). The phospho-mimetic Y148D, the siRNA-resistant wt-Aurora-A, phospho-depleted Y148F, phospho-depleted Aurora-A T288A and the double Aurora-A Y148F/T288A mutants were obtained through mutagenesis of Aurora-A-GFP using QuikChange Site-Directed mutagenesis kits (Agilent). The primers used in the mutagenesis reactions are showed in Supplementary Table 1. After transfection, the intracellular protein contents were analysed by SDS–polyacrylamide gel electrophoresis (SDS–PAGE) followed by western blotting, and the cells were further processed according to the experimental design. siRNA duplexes were transfected using Lipofectamine 2,000 (Invitrogen, Carlsbad, CA, USA), according to the manufacturer instructions. GM130 mRNA was targeted using siRNA duplexes directed against the sequence 5′-AAGTTAGAGATGACGGAACTC-3′ (Dharmacon; Lafayette, CA, USA). Non-targeting siRNA sequences were used as controls. Aurora-A siRNA and siRNA-resistant constructs were co-transfected using jetPRIME (Polyplus transfection, Illkirch, France), according to the manufacturer instructions. Aurora-A mRNA was targeted using siRNA duplexes directed against the sequence 5′-TTCTTAGACTGTATGGTTA-3′ (Dharmacon; Lafayette, CA, USA).

### Antibodies and reagents

Thymidine, BFA and fibronectin were from Sigma-Aldrich. Dimethylsulphoxide was from Carlo Erba (Italy). JNKi-VIII, PP2, digitonin and Mowiol were from Calbiochem. U0126 was from Promega, (USA); SU6656 was from Millipore (USA). Hoechst 33342, Alexa Fluor-conjugate secondary antibodies (1:400, A21202; A10037; A21206; A10042) and anti-golgin97 (A21270, 1:100) were from Molecular Probes (OR, USA). The primary antibodies were from the following sources: anti-phospho-histone H3 (pSer10) for western blotting (1:1,000) or for immunofluorescence (1:100; 06-570), anti-phospho-tyrosine (clone 4G10) (1:1,000; 05-321), anti-phospho-tyrosine Platinum agarose-conjugate (16–638) from Millipore (Billerica, MA, USA); anti-Histone H3 (1:1,000; sc-10809), anti-mouse (sc-2025) and anti-rabbit (sc-2027) pre-immune IgGs (1:5,000) from Santa Cruz; anti-GAPDH (1:80,000; 4,699–9,555) from AbD Serotech (USA); anti-Aurora-A (1:1,000; 610,938), and anti-GM130 (1:50; 610,823) from BD Biosciences (San Jose, CA, USA); anti-Src (clone 36D10, 1:1,000; 2,109), anti-Src-pY416 (clone D49G4, 1:1,000; 6,943) for western blotting, anti-Aurora-A (1:1,000; 3,092), anti-Aurora-pT288 (1:500; 3,079) from Cell Signaling; anti-Src-pY416 (1:100; 44-660A1) for immunofluorescence from Invitrogen; anti-Gal-T (1:250; HPA005729) and anti-γ-tubulin (1:200; T3559) from Sigma-Aldrich; anti-GFP (1,5μg per mg; clone 9F9.F9; Ab1218), anti-GFP (1:1,000: Ab290), anti-TGN46 (1:250; Ab50595), anti-giantin (1:500; Ab93281) and anti-PCNT (1:500; Ab28144) from Abcam; anti-α-Mannosidase II (1:150; HPA040627) from Atlas Antibodies (Stockholm, Sweden).

### Kinase assay

Aurora-A was expressed as intein–Aurora-A fusion protein in the BL21 (DE3) *Escherichia coli* strain. The soluble fraction from bacterial extracts was loaded on to a 10 ml chitin column (New England Biolabs). The bound fraction was treated with 100 mM DTT (dithiothreitol) to induce intein self-splicing^33^. A non-radioactive reaction was performed using 500 μg purified Aurora-A incubated with 50 ng Src (Enzo Life Sciences) in 50 μl kinase buffer (100 mM Tris-HCl, pH 7.2, 3 mM MgCl_2_, 1 mM NaF, 1 mM DTT) for 1 h at 37 °C in the presence of 1 mM ATP (Amersham Biosciences). Then, 1 μg recombinant His-tagged histone-H3 (Enzo Life Sciences) was added to the kinase reaction mix for 15 min at 37 °C. For the pre-incubation, 50 μM PP2 was added to Src for 10 min at 37 °C, before the addition of Aurora-A. Then the samples were analysed by SDS–PAGE.

### Immunoprecipitation

For each immunoprecipitation, HeLa cells were lysed after reaching 80% confluency, using 500 μl ice-cold extraction buffer containing 20 mM HEPES, pH 7.5, 10 mM NaCl, 15 mM MgCl_2_, 10 mM NaF, 1 mM EDTA, 1 mM Na_3_VO_4_, 0.5% (w/v) Nonidet-P-40, supplemented with protease and phosphatase inhibitors cocktail (Roche). After 30 min of cell lysis on ice in the extraction buffer, the samples were centrifuged at 6,000*g* for 30 min at 4 °C. The supernatants were then normalized to 500 μg and incubated under rotation at 4 °C with 1.3 μg anti-GFP antibody, as indicated for 2 h or O/N, before addition of protein A/G-Sepharose for a further 30 min. When using the anti-phospho-tyrosine antibody conjugated to the agarose matrix (4G10 Platinum, Millipore), the resin was first equilibrated in extraction buffer and then 20 μl of resuspended resin was used for each sample, incubating O/N on a rotating shaker. The beads were collected and washed twice with 500 μl ice-cold complete extraction buffer, and twice with 500 μl ice-cold extraction buffer without detergent. The western blots were visualized using the ECL reagents (GE Healthcare), according to the manufacturer's instructions for ECL-based detection. Where stated, band intensities were quantified using Quantity One software (Bio-Rad Laboratories). Full-scan images of all western blotting data are reported in Supplementary Fig. 6.

### Immunofluorescence

HeLa cells were grown on glass coverslips coated with 10 μg ml^−1^ fibronectin and treated according to the experimental procedure. They were then either fixed with 4% pre-warmed paraformaldehyde for 10 min, at RT, or with ice-cold methanol for 5 min, at −20 °C. They were then washed once (for paraformaldehyde fixing) or three times (for methanol fixing) in PBS. The blocking reagent (0.05% saponin, 0.5% BSA, 50 mM NH_4_Cl) was then added to the cells for 20 min, followed by a 1 h or O/N incubation with the primary antibodies in the blocking reagent. The cells were then washed with PBS and incubated with the secondary antibodies (1:400) with 2 μg ml^−1^ Hoechst33342, for 45 min. The coverslips were then mounted on glass microscope slides with Mowiol. Immunofluorescence samples were examined using a confocal laser microscope (Zeiss LSM700 confocal microscope system; Carl Zeiss, Gottingen, Germany) equipped with 63 × 1.4 NA oil objective. Optical confocal sections were taken at 1 Airy unit, with a resolution of 512 × 512 pixels or 1,024 × 1,024 pixels, and exported as .JPEG or .TIF files. The images were cropped with Adobe Photoshop CS6 and composed using Adobe Illustrator CS6. For the staining of pTyr416-Src at the Golgi, HeLa cells were grown on 10 μg ml^−1^ fibronectin-coated glass coverslips and treated according to the experimental procedure. Before fixing with 4% paraformaldehyde, the cells were washed twice with ice-cold KHM buffer (125 mM CH_3_CO_2_K, 25 mM HEPES pH 7.2, 2.5 mM Mg(CH_3_COO)_2_), then permeabilized by incubating the coverslips on ice in 0.5 ml KHM containing 30 μg ml^−1^ digitonin, for 5 min, and finally washed for 7 min at RT with KHM buffer. The cells were subsequently fixed and processed for immunofluorescence microscopy. To visualize and quantify γ-tubulin and/or PCNT staining for the maturation of the centrosome in the mentioned experiments (see Fig. 6 and Supplementary Fig. 5c–d), a specific setting of the confocal microscope was applied in order to avoid ‘pixel saturation'. For the experiments in which γ-tubulin and/or PCNT were used as markers of centrosome and did not require quantification (see Fig. 1a and Supplementary Figs 1a,4b and 5b), the setting was applied with an increased gain value without considering the ‘pixel saturation' for the marker.

### ‘Golgi object' assays

HeLa cells arrested in S-phase using the double-thymidine block were either fixed in S-phase, or treated for 18 h with bis-benzimide, for G2-arrest, or for 6 h with 10 μM U0126, or for 2 h with 10 μM SU6656. For quantitative analysis of the Golgi integrity (number of objects per Golgi), the Golgi-associated fluorescence of randomly chosen cells was acquired. The freeware ImageJ software package was used to automatically apply a threshold and to count the above-threshold fluorescent ‘Golgi objects', using the ‘Analyse Particle' function. All of the images were acquired at maximal resolution, under fixed-imaging conditions. For qualitative analysis of the phenotype of the Golgi apparatus, the GM130 staining was processed with the freeware ImageJ software package using the ‘Invert' function.

### Proximity ligation assay

Proximity ligation assays were performed using the Duolink anti-Mouse MINUS and anti-Rabbit PLUS *in situ* PLA probes and the Duolink *in situ* Detection Reagents Red (Olink Bioscience), following the manufacturer's instructions. The amplified signals were analysed using laser scanning confocal microscopy.

### Golgi fragmentation assay in permeabilised NRK cells

NRK cells were grown to 60% confluency on fibronectin-coated glass coverslips and incubated with 2 mM thymidine for 8 h to 10 h at 37 °C. The cells were then washed at RT in KHM buffer and rapidly shifted onto ice for 2 min. The cells were then permeabilized with 30 μg ml^−1^ digitonin in KHM buffer, and washed with KHM buffer also containing 1M KCl. The cells were incubated on 50 μl reaction mixture, upside down at 32 °C for 1 h. The reaction mixture contained KHM or mitotic extract (final concentration, 9.5 mg ml^−1^) and an ATP-regeneration system. When stated, the permeabilized cells were incubated with mitotic cytosol in the presence of 10 μM SU6656. Cells were fixed and processed for immunofluorescence using an anti-giantin antibody to visualise the structure of the Golgi apparatus. To prepare mitotic cytosol, NRK cells were treated with 2 mM thymidine for 10–12 h to arrest cells in S-phase. Then, the cells were washed and incubated O/N with 500 ng ml^−1^ nocodazole. The rounded mitotic cells were harvested and lysed in lysis buffer (15 mM PIPES (pH 7.4), 50 mM KCl, 10 mMMgCl_2_, 20 mM β-mercaptoethanol, 20 mM β-glycerophosphate, 15 mM EGTA, 0.5 mM spermidine, 0.2 mM spermine, 1 mM DTT and protease inhibitors). Interphase cytosol was prepared from NRK cells arrested in S-phase by O/N treatment with 2 mM thymidine^21^.

### Phosphoproteomic analysis

Protein immunoprecipitates were separated on 12% T SDS–PAGE gels, which were then stained with colloidal Coomassie G-250 (ref. 53). Samples of the Aurora-A protein bands (at 45 kDa) were manually excised from the gels, in-gel reduced with 10 mM DTT in 100 mM NH_4_HCO_3_ (30 min, at 56 °C) and S-alkylated with 55 mM iodoacetamide in 100 mM NH_4_HCO_3_ (20 min, at room temperature, in the dark)^54^. Gel particles were washed, dried and rehydrated with the digestion solution (12.5 ng μl^−1^ of trypsin in 50 mM NH_4_HCO_3_, 5 mM CaCl_2_). After incubation at 5 °C (45 min), the supernatant was replaced by 50 mM NH_4_HCO_3_, 5 mM CaCl_2_, and gel particles were incubated overnight, at 37 °C. Phosphopeptides were enriched from whole protein digests using IMAC disc columns and TiO_2_ microcolumns (55). In the first case, peptide digests solved in 5% acetic acid were loaded on SwellGel Ga-chelated discs columns (Pierce), incubated for 5 min, at 25 °C, centrifuged at 5,000 r.p.m. and washed with increasing amounts of acetonitrile in 0.1% acetic acid and finally with water^55^. Phosphopeptides were initially eluted with 100 mM NH_4_HCO_3_, pH 8 and finally with 50% acetonitrile in 100 mM NH_4_HCO_3_, pH 8. Eluted solutions were mixed and analysed for phosphopeptides by nanoLC-ESI-LIT-MS/MS. In the second case, TiO_2_ columns were assembled by using as plug C_8_ material from a 3 M Empore C_8_ extraction disk, which was then covered with TiO_2_ beads (GL Science, Inc., Japan) suspended in 80% acetonitrile, 0.1% trifluoracetic acid (TFA). Columns were washed with 80% acetonitrile, 0.1% TFA before sample loading. Peptide samples were solved in loading buffer (80% acetonitrile, 0.1% TFA containing 20 mg ml^−1^ 2,5-dihydroxybenzoic acid) and then loaded. TiO_2_ columns were washed with loading buffer and subsequently with 80% acetonitrile, 0.1% TFA. Bound phosphopeptides were eluted using 10 μl of NH_4_OH (pH 10.5) and then analysed by nanoLC-ESI-LIT-MS/MS. Samples were analysed by nanoLC-ESI-LIT-MS/MS using a LTQ XL mass spectrometer (Thermo Finnigan, San Jose, CA, USA) equipped with a Proxeon nanospray source connected to an Easy-nanoLC (Proxeon, Odense, Denmark). Peptide mixtures were separated on an Easy C18 column (10 × 0.075 mm, 3 μm) (Proxeon, Odense, Denmark), using a linear gradient from 5 to 40% acetonitrile in 0.1% formic acid, over 60 min, at a flow rate of 300 nl min^−1^. Spectra were acquired the range *m/z* 400–2,000. Acquisition was controlled by a data-dependent product ion-scanning procedure over the three most abundant ions, enabling dynamic exclusion (repeat count 1 and exclusion duration 1 min). The mass isolation window and collision energy were set to *m/z* 3 and 35%, respectively. Analysis was performed in duplicate. The proteome Discoverer 1.4 platform (Thermo, San Jose, CA, USA) including the Mascot 2.3 algorithm (Matrix Science, UK) was used to identify Aurora-A phosphopeptides/peptides from nano LC-ESI-LIT-MS/MS data. This software was run against an indexed database that contained human Aurora-A, and common contaminants and protease sequences. Cyscarbamidomethylation was used as a fixed modification, and Met oxidation and Ser/Thr/Tyr phosphorylation as differential modifications. Mass tolerance values of *m*/*z* 2.0 and 0.8 were used for parent and fragment ions, respectively, and trypsin as a proteolytic enzyme. Candidates with a Mascot value>25 were further evaluated by visual inspection of the fragmentation spectra. GPMAW 4.23 (Lighthouse, Denmark) software was also used to confirm peptide identification by MS/MS (tandem mass spectrometry) data.

### Data availability

The authors declare that the data supporting the findings of this study are available within the article (and its Supplementary Information files) and are available upon request.

## Additional information

**How to cite this article:** Barretta, M. L. *et al*. Aurora-A recruitment and centrosomal maturation are regulated by a Golgi-activated pool of Src during G2. *Nat. Commun.* 7:11727 doi: 10.1038/ncomms11727 (2016).

## Supplementary Material

Supplementary Information Supplementary Figures 1-6 and Supplementary Table 1.
