# Supplementary material for: Aurora-A recruitment and centrosomal maturation are regulated by a Golgi-activated pool of Src during G2
Source: Nat Commun. 2016 May 31;7:11727. doi: 10.1038/ncomms11727 (PMC4895030; doi:10.1038/ncomms11727)
Supplement: Supplementary Information — Supplementary Figures 1-6 and Supplementary Table 1. [file ncomms11727-s1.pdf]

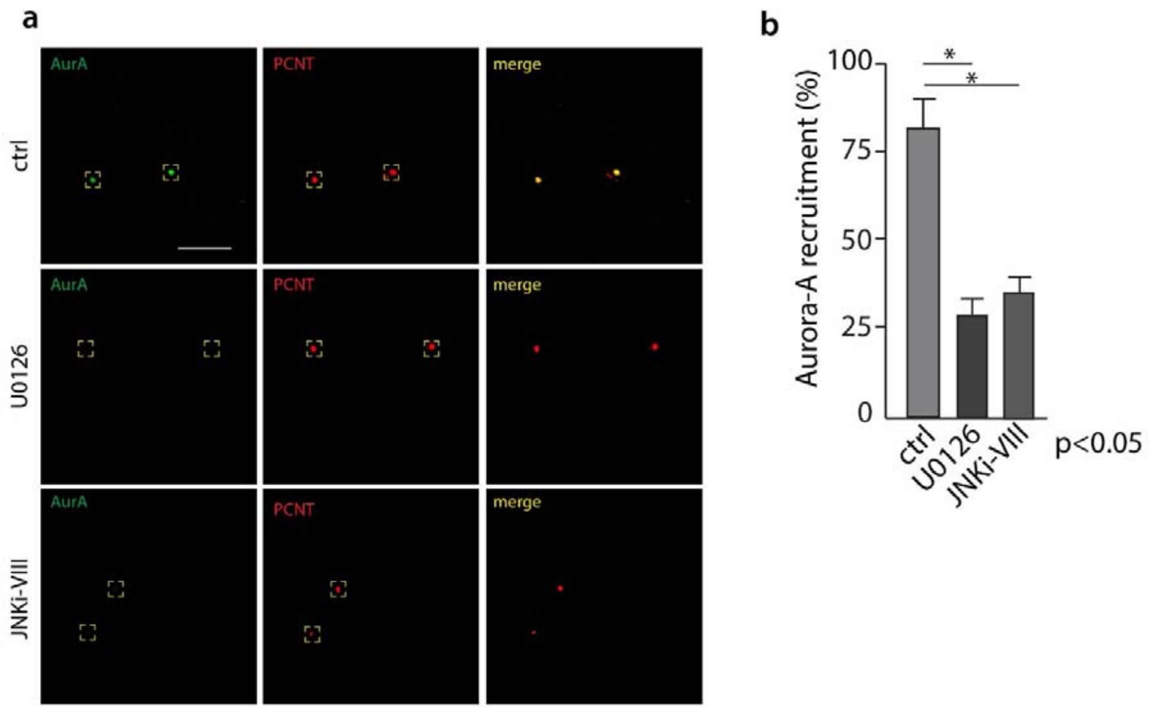

Supplementary Figure 1: The block of MEK and JNK2 pathways prevent the recruitment of Aurora-A to the centrosome

(a) Representative images of HeLa cells grown on coverslips and arrested in S-phase using the double-thymidine block. 6h before fixing, the cells were incubated with 80µM U0126 (MEK inhibitor) or for 2h with 50µM JNKi-VIII or with DMSO as control. After 8h without thymidine, the cells were processed for immunofluorescence confocal microscopy with, an anti-Aurora-A antibody and an anti-PCNT antibody as reference for the centrosome. **(b)** Quantification of Aurora-A recruitment to the centrosome of HeLa cells treated as in (a). Two-tailed Student *t*-tests were applied to the data (P-value <0.05).

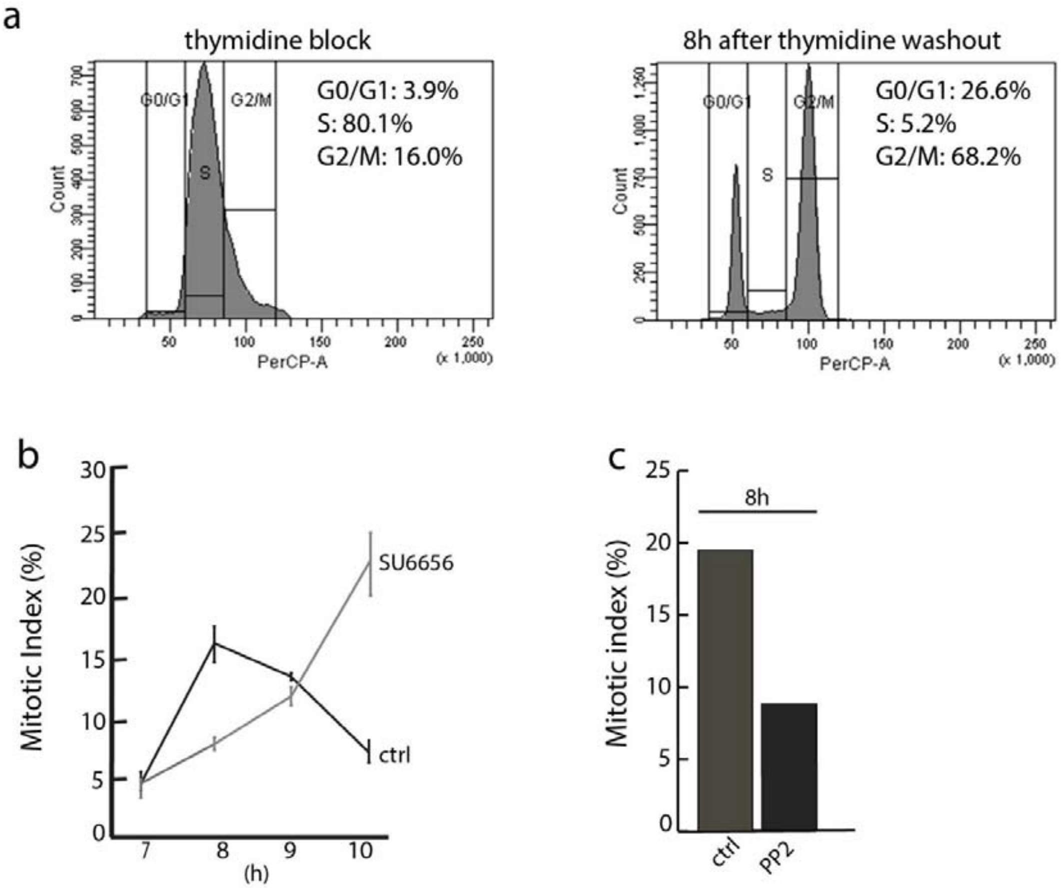

Supplementary Figure 2: Src inhibition delays the normal timing of cell-cycle progression

(**a**) HeLa cells were arrested in S-phase by double synchronization with the sequential addition of 2 mM thymidine. The FACS analyses of S-phase blocked cells (left panel) or G2/M enriched cells (8h after thymidine washout - right panel) are shown. **(b-c)** Synchronized HeLa were incubated with the Src inhibitors PP2 (10 µM) and SU6656 (10 µM) for 2 h before the fixing. The cells were processed for immunofluorescence with 2 µg/ml Hoechst 33342 for the cell-cycle phase. Data are means (±s.d.) from four independent experiments, each carried out in duplicate. More than 200 cells were counted for each condition.

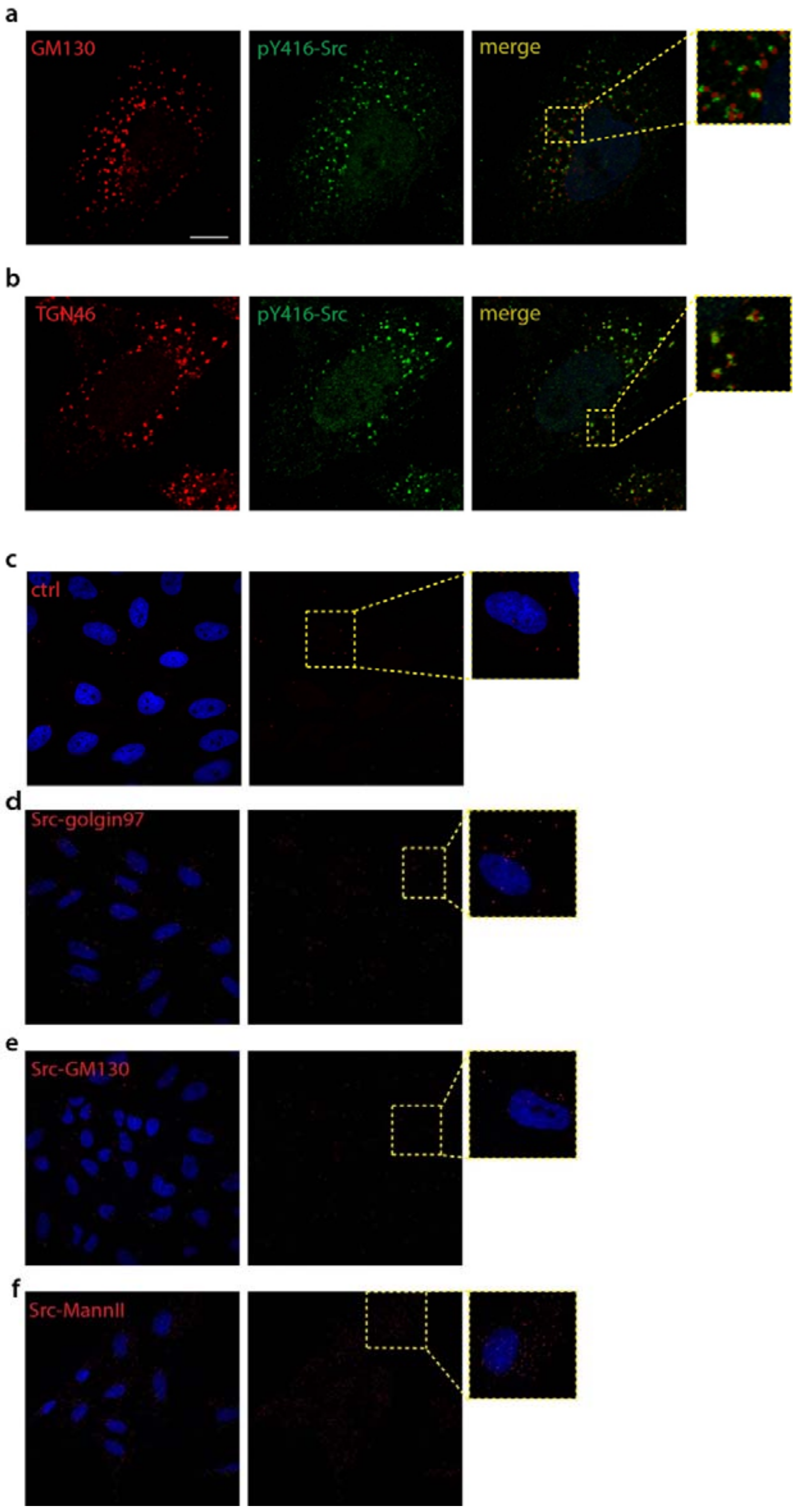

Supplementary Figure 3: Active Src localizes at the TGN.
(a-b) HeLa cells were treated with 33μM nocodazole for 3h and active Src was monitored using an anti pY416-Src antibody. The Golgi-specific compartment was decorated with GM130 (red, a) or TGN-46 (red, b). Scale bar: 7.5 μM. **(c-d-e-f)** Representative images of the PLA performed on HeLa cells using only secondary probes (c) or anti-Src antibody in combination with anti-golgin97 (d), anti-GM130 (e) or anti-Mannosidase II (f) antibodies.

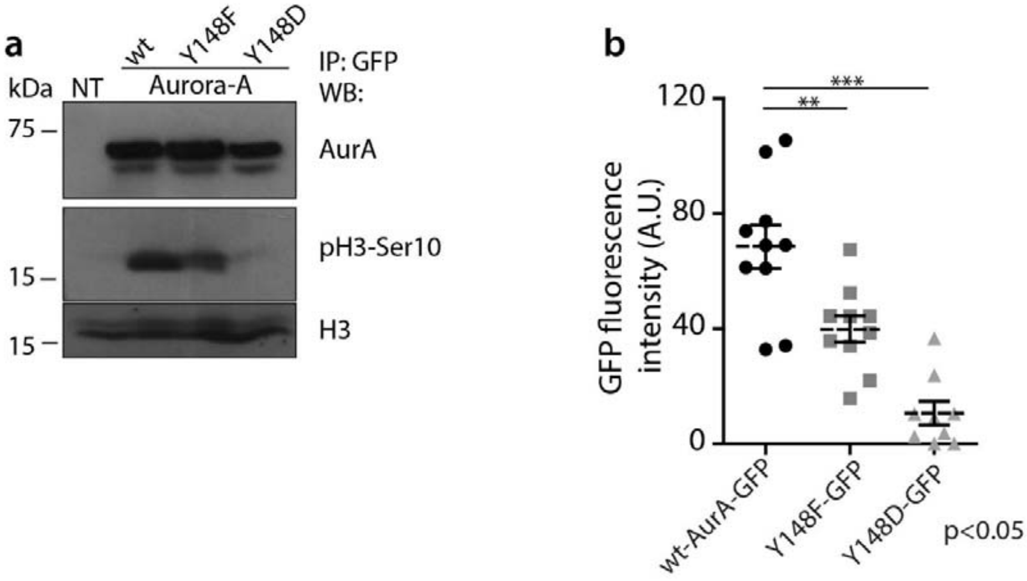

Supplementary Figure 4: Aurora-A catalytic activity and centrosomal recruitment are impaired when Tyr148 is replaced by Phe.

(a) Western Blot of a kinase assay performed using immunoprecipitated Aurora-A from synchronized HeLa cells that were transfected for 24h with GFP-vectors expressing wt, Y148F or Y148D-Aurora-A. The *in vitro* kinase assay was performed using a fixed amount of recombinant purified His-Histone-H3 as substrate to evaluate Aurora-A catalytic activity. **(b)** Cells overexpressing wt, Y148F or Y148D-Aurora-A-GFP were processed for immunofluorescence to measure the fluorescence intensity of the recruited Aurora-A recombinant proteins. Anti-γ-tubulin antibody was used as reference for the centrosome (A.U.: arbitrary units). All images for the quantification were acquired at maximal resolution, under fixed imaging conditions. Equal circle areas were used to select the centrosomes regions and a non-centrosome region with a similar background. Quantification data are means (±s.d.) from two independent experiments. Two-tailed Student *t*-tests were applied to the data (p-value <0.05).

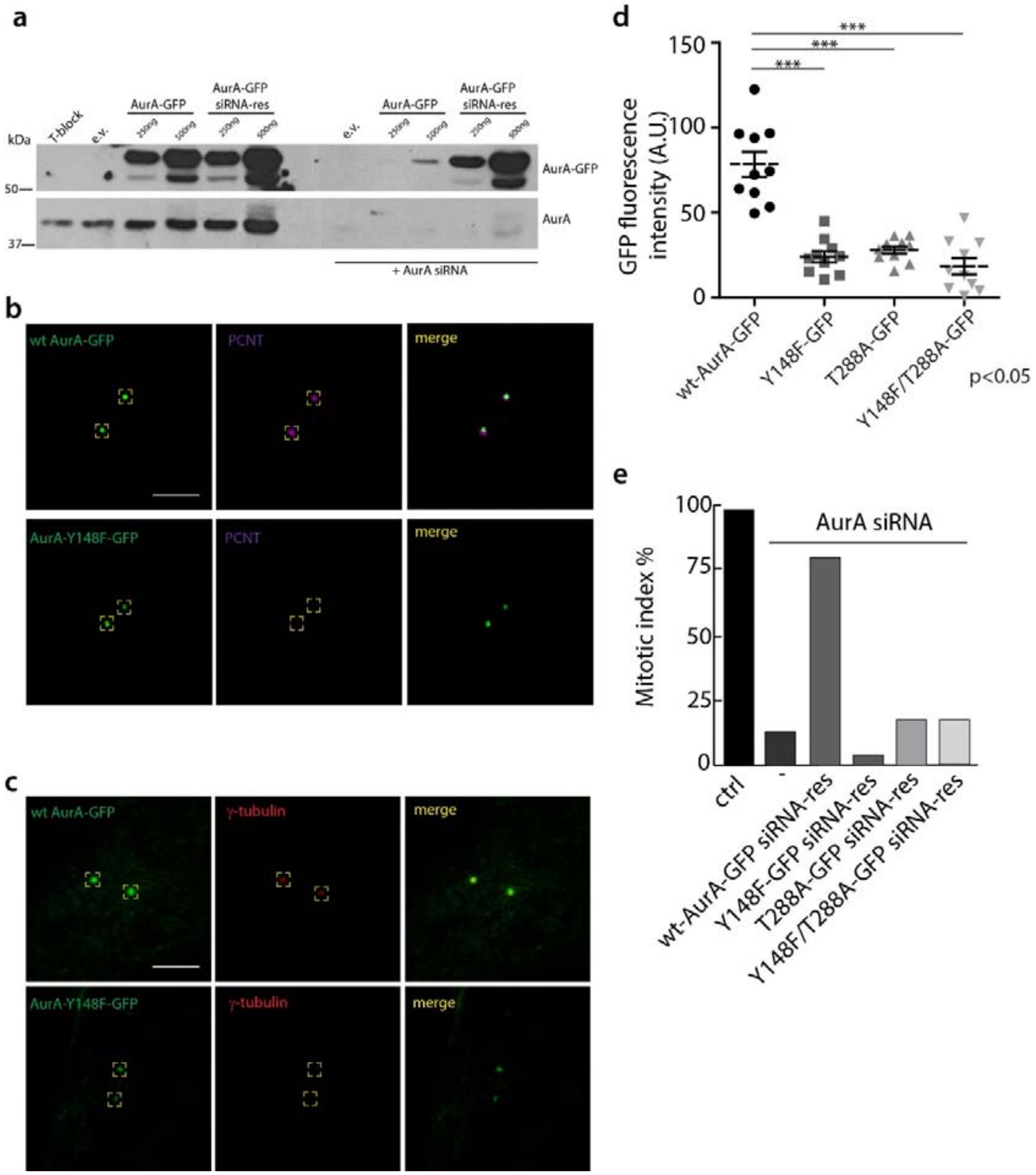

Supplementary Figure 5: Effect of Aurora-A mutants on centrosomal maturation

(a) Western Blot of synchronized HeLa cells transfected with Aurora-A or Aurora-A siRNA-resistant mutants in presence or in absence of the specific siRNA for endogenous Aurora-A. **(b-c)** Representative images of the cells quantified in Fig 6c and 6d referred to wt protein or Aurora-A-Y148F. Scale bars: 5µM. **(d)** HeLa cells were transfected for 24h with wt-Aurora-A or Aurora-A mutants (Y148F, T288A or Y148F/T288A) tagged with a GFP reporter. Cells were processed for immunofluorescence to measure the fluorescence intensity of the recruited GFP and an anti-γ-tubulin antibody was used as reference for the centrosome (A.U.: arbitrary units). All images for the quantification were acquired at maximal resolution, under fixed imaging conditions. Equal circle areas were used to select the centrosomes regions and a non-centrosome region with a similar background. Quantification data are means (± s.d.) from three independent experiments. Two-tailed Student *t*-tests were applied to the data ($p<0.05$). **(e)** Mitotic index of synchronized HeLa cells transfected with Aurora-A or Aurora-A siRNA-resistant mutants in presence of the specific siRNA for endogenous Aurora-A. Quantification data are means from two independent experiments. Data are normalized to control untreated cells (ctrl).

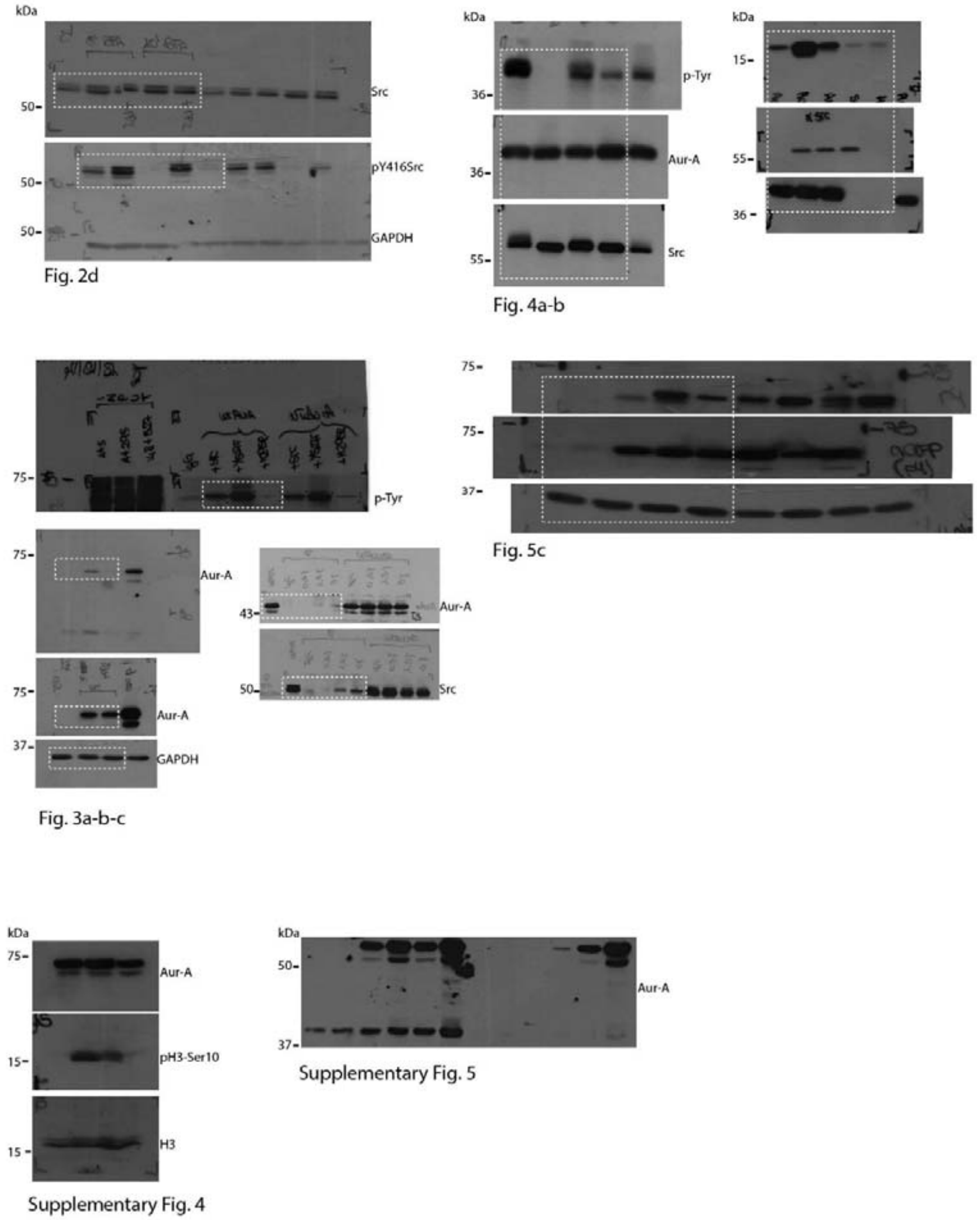

Supplementary Figure 6: Full scan images of all Western blotting data.

siRNA Aur-A res for	5'-CCCACCTTCGGCATCCTAATATTCTTCGGTTATACGGCTATTTCCATGATGCTACCAGAGTCTACC-3'
siRNA Aur-A res rev	5'-GGTAGACTCTGGTAGCATCATGGAAATAGCCGTATAACCGAAGAATATTAGGATGCCGAAGGTGGG-3'
hAur-A T288A for	5'-CCATCTTCCAGGAGGACCGCTCTCTGTGGCACCCTGG-3'
hAur-A T288A rev	5'-CCAGGGTGCCACAGAGAGCGGTCCTCCTGGAAGATGG-3'
hAur-A Y148F for	5'-CTTTTCTCTTGCCAAAAAAACATTACCAAACTTTCCTTTACCCAGA-3'
hAur-A Y148F rev	5'-TCTGGGTAAAGGAAAGTTTGGTAATGTTTTTTTGGCAAGAGAAAAG-3'
hAur-A Y148D for	5'-GCTTTGCTTTTCTCTTGCCAAATCAACATTACCAAACTTTCCTTTAC-3'
hAur-A Y148D rev	5'-GTAAAGGAAAGTTTGGTAATGTTGATTTGGCAAGAGAAAAGCAAAGC-3'

Supplementary Table 1: Primer sequences used for mutations of Aurora-A
